# Sirt5 Inhibits BmNPV Replication by Promoting a Relish-Mediated Antiviral Pathway in *Bombyx mori*

**DOI:** 10.3389/fimmu.2022.906738

**Published:** 2022-05-23

**Authors:** Mengmeng Zhang, Shigang Fei, Junming Xia, Yeyuan Wang, Hongyun Wu, Xian Li, Yiyao Guo, Luc Swevers, Jingchen Sun, Min Feng

**Affiliations:** ^1^ Guangdong Provincial Key Laboratory of Agro-Animal Genomics and Molecular Breeding, College of Animal Science, South China Agricultural University, Guangzhou, China; ^2^ Insect Molecular Genetics and Biotechnology, National Centre for Scientific Research Demokritos, Institute of Biosciences and Applications, Athens, Greece

**Keywords:** sirtuins, Sirt5, *Bombyx mori*, BmNPV, Relish

## Abstract

Silent information regulators (Sirtuins) belong to the family of nicotinamide adenine dinucleotide (NAD^+^)-dependent histone deacetylases (HDACs) that have diverse functions in cells. Mammalian Sirtuins have seven isoforms (Sirt1–7) which have been found to play a role in viral replication. However, Sirtuin members of insects are very different from mammals, and the function of insect Sirtuins in regulating virus replication is unclear. The silkworm, *Bombyx mori*, as a model species of Lepidoptera, is also an important economical insect. *B. mori* nucleopolyhedrovirus (BmNPV) is a major pathogen that specifically infects silkworms and causes serious losses in the sericulture industry. Here, we used the infection of the silkworm by BmNPV as a model to explore the effect of Sirtuins on virus replication. We initially knocked down all silkworm Sirtuins, and then infected with BmNPV to analyze its replication. Sirt2 and Sirt5 were found to have potential antiviral functions in the silkworm. We further confirmed the antiviral function of silkworm Sirt5 through its effects on viral titers during both knockdown and overexpression experiments. Additionally, Suramin, a Sirt5 inhibitor, was found to promote BmNPV replication. In terms of molecular mechanism, it was found that silkworm Sirt5 might promote the immune pathway mediated by Relish, thereby enhancing the host antiviral response. This study is the first to explore the role of Sirtuins in insect-virus interactions, providing new insights into the functional role of members of the insect Sirtuin family.

## Introduction

Silent information regulators (Sirtuins) are a family of nicotinamide adenine dinucleotide (NAD)^+^-dependent histone deacetylases (HDACs) (1). In addition to having deacetylase activity, some Sirtuins have alternative enzymatic activities including ADP ribosyltransferase (Sirt4 and Sirt6), demalonylase and deglutarylase (Sirt5), desuccinylase (Sirt5 and Sirt7), and demyristoylase (Sirt6) (2, 3). Due to the different enzymatic activities and target substrates, Sirtuins are essential for many biological processes including cell survival, anti-aging, DNA repair, proliferation, inflammation, mitochondrial energy homeostasis, and metabolism (1, 3–5).

Studies on Sirtuin biology have made great progress in the past two decades, mainly emphasizing the importance of these enzymes in human biology and disease (4). However, there are few reports on the regulation of viral replication by Sirtuins (6). In particular, there is increasing evidence that Sirtuins are associated with the persistence and pathogenesis of viral infections caused by human immunodeficiency virus, influenza A virus, herpes simplex virus 1, and human papilloma virus (2, 3). Host metabolism regulated by Sirtuins plays a key role in virus-host interaction (7, 8). Another mechanism by which Sirtuins control viruses may be the modulation of the expression of viral genes and viral replication (7). Studies have shown that overexpression of the type 5 isoenzyme of Sirt2 inhibited hepatitis B virus (HBV) replication (9). It was found that Sirt3 by way of its acetylation function can inhibit the production of human cytomegalovirus (HCMV) (10). Recently, a research team found that Sirt5 interacted with the DEAD-box polypeptide 3 (DDX3) and demalonylated DDX3, which was critical for TANK-binding protein kinase 1 (TBK1)-IFN regulatory factor 3 (IRF3) activation, and promoted innate immune responses against RNA and DNA viruses (5). Indeed, according to the existing research reports, there are various ways by which Sirtuins can affect the process of virus-host interactions.

Mammals have seven distinct members in the Sirtuin family named Sirt1, Sirt2, Sirt3, Sirt4, Sirt5, Sirt6 and Sirt7. Sirt1 predominantly locates in the nuclear compartment but also has roles in the cytoplasm (11, 12). Sirt2 is found in the cytosol, while Sirt3, Sirt4 and Sirt5 are mitochondrial proteins (13). Both Sirt6 and Sirt7 have a nuclear localization. However, Sirtuin family members in invertebrates differ considerably from Sirtuins in mammals. Genomes of ecdysozoan species (that grow by molting and include nematodes and arthropods) do not encode a Sirtuin of the Sirt3 group, which must have been lost prior to or early in their evolution (14). Fully sequenced *Caenorhabditis elegans* and *C. briggsae* genomes (15) contain Sirt1 and Sirt4 homologs, while an additional divergent Sirtuin in both nematode species does not cluster with any other Sirtuin groups. Interestingly, another nematode *Brugia malayi* does not contain a Sirt4 homolog, although Sirt1, Sirt6 and Sirt7 homologs are present (16). Within arthropods, several insects including the honey bee *Apis mellifera* (17), the parasitoid wasp *Nasonia vitripennis*, and the red flour beetle *Tribolium castaneum* (18) contain homologs of all Sirtuins except Sirt3 (14). The genome of *Drosophila melanogaster* was found lacking of a Sirt5 homolog (14). Thus, the loss of specific Sirtuin members is characteristic of nematodes and arthropod lineages (14). The differences between the members of the Sirtuin family in mammals and insects have attracted our interest, especially with respect to the role of Sirtuins in viral infection. In fact, to our knowledge, there are no reports on the regulation of virus infections in insects by Sirtuin proteins.

*Bombyx mori*, as the model species of Lepidoptera and the only truly domesticated insect, has been used extensively in basic research. In particular, silkworm disease caused by *B. mori* nucleopolyhedrovirus (BmNPV) has been studied in detail because of its serious impact in sericulture. BmNPV specifically infects *B. mori* and belongs to the family of *Baculoviridae*, a group of enveloped insect-specific viruses with large circular DNA genomes that contain 120-170 open reading frames (19). During the baculovirus infection cycle, two types of virions are produced. Occlusion-derived viruses (ODVs) become incorporated in polyhedra or occlusion bodies and are responsible for the spread in the environment and the infection of new hosts after feeding. The other virion type is the budding virus (BV), which is mainly responsible for systemic infection in the host insect, leading to secondary infection of internal tissues (20). It was reported that only 5 Sirtuins (Sirt2, Sirt4, Sirt5, Sirt6 and Sirt7) exist in the silkworm genome (21). Until now, the role of Sirtuins during BmNPV infection has not been investigated. Since the BmNPV-silkworm infection model is very well established, it is considered very suitable for the study of the role of Sirtuins in insect-virus interactions.

In this study for the first time the function of Sirtuins in the regulation of baculovirus infections was explored for the first time. It was found that BmSirt5 can inhibit the replication of BmNPV. The underlying mechanism may be that BmSirt5 affects the function of the central immune response transcription factor Relish to stimulate the antiviral defense.

## Materials And Methods

### Cell Culture and Virus

The ovary-derived cell line of *B. mori* (BmN cells) (22, 23) was maintained at 28°C in Grace medium supplemented with 10% fetal bovine serum (FBS) (Gibco, USA). The larvae of the silkworm strain Dazao were reared with fresh mulberry leaves at 28°C and 70-80% relative humidity. The recombinant BmNPV-eGFP was constructed using the BmNPV-based Bac-to-Bac System (*B. mori* MultiBac) (24) and kept in the Guangdong Provincial Key Laboratory of Agro-animal Genomics and Molecular Breeding.

### Bioinformatics Analysis of the Silkworm Sirtuin Family Genes

The chromosomal localization of the silkworm Sirtuin family genes was determined based on the genome of the domesticated silkworm from SilkDB 3.0 (https://silkdb.bioinfotoolkits.net/main/species-info/) (25). Predictive analysis of cellular localization of silkworm Sirtuin family genes was carried out with LocTree3 (https://www.rostlab.org/services/loctree3/) (26). Based on the sequences of Sirtuins obtained from the National Center for Biotechnology Information (NCBI), the phylogenetic analysis was performed with Molecular Evolutionary Genetics Analysis (MEGA) Software Version 7.0 using the neighbor-joining analysis method (NJ), based on 1000 repeats.

### Quantitative Reverse Transcription PCR (qRT-PCR) and Reverse Transcription PCR (RT-PCR)

Total RNA of BmN cells or different tissues was extracted by Kit RNA fast 2000 (Fastagen, China) and reverse transcribed to cDNA by the RT reagent kit with gDNA Eraser (TaKaRa, Japan). qRT-PCR tests were conducted using the specific primers listed in **Table S1**. The silkworm *Rp49* gene was used as reference gene. qRT-PCR was performed on the Bio-Rad CFX96 Real-Time Detection System using iTaqTM Universal SYBR^®^ Green Supermix Kit reagents (Bio-Rad, USA). Calculation of relative levels of mRNA expression was performed using the 2^-ΔΔCt^ method. Semi-quantitative RT-PCR was used to detect the relative mRNA expression of *BmSirt7* using the silkworm *Rp49* gene as an internal control.

### Viral Titer Assay

To evaluate the replication of BmNPV at 24, 48 and 72 h post-infection (hpi), cell supernatants were collected at 24, 48 and 72 hpi and diluted serially by 10-fold. Then, 10 µL of each dilution was added to 96-well plates (Thermo, USA) containing 90 µL of BmN cells (1.25×10^4^ cells/well). Green fluorescence was recorded at 72 hpi. The 50% tissue culture infectious dose (TCID_50_) was calculated according to the method of Reed and Muench (27). In experiments that required synchronous infection, BmN cells were first incubated with virus for 1 h and then replaced with fresh Grace medium containing 10% FBS. This time point was defined as 0 hpi.

### Detection of Sirtuin mRNA Expression After BmNPV Infection in the Silkworm

Newly molted fifth-instar silkworm larvae divided into two groups were injected with either 10 µL of BmNPV-eGFP (10^5.8^TCID_50_/0.1 mL) or PBS (Negative Control, NC). Fat body tissue collected from three silkworms were pooled together as one repeat sample at 24, 48 and 72 hpi. The midgut and hemocyte samples were also collected at these time points. Each group contained 3 replicates (3 larvae/replicate). Sirtuin mRNA expression in BmNPV-infected and PBS-treated silkworms was detected by qRT-PCR.

### Double Strand RNA Synthesis

Double strand RNA (dsRNA) was synthesized using the T7 RiboMAX™ Express RNAi System (Promega, USA) according to the manufacturer’s instructions. DsRNA-DsRed was synthesized as negative control. All primers used for the dsRNA synthesis are listed in **Table S1**.

### Knockdown of Silkworm Sirtuin Genes and Measurement of BmNPV Replication in BmN Cells

BmN cells (1.25×10^5^ cells/well) were cultured in 24-well cell plates (Thermo, USA) and transfected with dsRNA targeting *BmSirt2*, *BmSirt4*, *BmSirt5* and *BmSirt6* (5 μg/well) using the FuGENE HD transfection reagent (Promega, USA). BmN cells transfected with dsRNA-DsRed (5 μg/well) were used as control. At 24 h post-transfection, BmN cells were infected with BmNPV-eGFP at 1 MOI. The expression of the viral gene *Vp39* was detected by qRT-PCR at 24, 48 and 72 hpi. For the experimental group with knock-down of *BmSirt5*, additional measurements of virus titer and green fluorescence intensity were employed to further evaluate the replication of BmNPV-eGFP.

### Overexpression of BmSirt5 and Detection of BmNPV Replication in BmN Cells

For overexpression of BmSirt5 (fused with 3×Flag tag), the PCR-amplified *BmSirt5* coding sequence from BmN cells was cloned into the pIEX plasmid (MiaoLingPlasmid, Wuhan, China) using the restriction enzyme sites of *Sac* I and *Sal* I. The specific primers used to amplify *BmSirt5* are shown in **Table S1**. BmN cells (1.25×10^5^ cells/well) were transfected with pIEX-BmSirt5 plasmid (500 ng/well) using FuGENE HD transfection reagent according to the manufacturer’s instructions (Promega, USA). pIEX-eGFP was used as the control. Western blotting was employed to detect protein expression of BmSirt5, eGFP and Tubulin at 24, 48, 72 and 96 h post-transfection using mouse anti-flag antibody (Beyotime, China), mouse anti-GFP antibody (Beyotime, China) and rabbit anti-tubulin antibody (Beyotime, China), respectively.

At 24 h post-transfection, BmN cells were infected with BmNPV-eGFP (1 MOI). Cell total RNA and supernatants were collected at 24, 48 and 72 hpi to detect the replication of BmNPV using qRT-PCR and viral titer assay.

### Suramin Treatment of BmN Cells

Suramin as a compound that inhibits Sirt5 NAD^+^-dependent deacetylase activity (28). To determine the optimal concentration of Suramin, BmN cells (1.25×10^4^ cells/well) were pre-incubated with a gradient of increasing concentrations of Suramin (5, 10, 15, 20, 25, 30 and 35 μM, CHEMEGEN, USA) for 24 h in 96 well-plates. Cytotoxicity was determined using Cell Counting Kit-8 dye (Beyotime, China). The optimum concentration was considered the maximal concentration at which Suramin did not cause measurable toxicity to the cells. In all consecutive experiments, BmN cells (1.25×10^5^ cells/well) were pretreated with Suramin at the optimum concentration (30 μM) for 12 h. Cells pretreated with the same volume of solvent (ddH_2_O) were used as control. BmN cells pretreated with Suramin or ddH_2_O were infected with BmNPV at 1 MOI for 1 h. Then, the supernatant was replaced with Grace medium (10% FBS) supplemented with Suramin (at 30 μM). Total RNA and cell supernatants were collected at 24, 48 and 72 hpi to detect BmNPV replication using qRT-PCR and viral titer assay.

### Suramin Treatment of Silkworm

According to the manufacturer’s instructions, newly molted fifth-instar silkworm larvae (about 0.3 g in weight) divided into two groups were injected with either Suramin (3 μg in 2.3 μL of ddH_2_O) or ddH_2_O (NC, 2.3 µL). Twelve hours later, silkworms pretreated with Suramin or ddH_2_O were injected with BmNPV-eGFP (10^5.8^TCID_50_/0.1 mL, 7.7 μL/larva) together with Suramin (2^nd^ dose of 3 μg) or ddH_2_O (2.3 μL/larva). This time point was defined as 0 hpi. Fat bodies from Suramin-treated and control groups were collected at 24, 48 and 72 hpi. For every time point, three animals were pooled together. Each group contained 3 replicates (3 larvae/replicate). The expression of the viral gene *Vp39* in fat body tissue was detected by qRT-PCR.

### Quantification of Expression of the Immune Genes BmRelish, CecA and CecB

Fat body and BmN cell samples collected from BmNPV-infected and uninfected groups at 24, 48 and 72 hpi were used to detect the transcriptional level of *BmRelish*, *CecA* and *CecB* by qRT-PCR. BmN cells (1.25×10^5^ cells/well) transfected with dsRNA-BmSirt5 (5 μg/well), pIEX-BmSirt5 plasmid (500 ng/well) or treated with Suramin (30 μM) were infected with BmNPV-eGFP (1 MOI) at 24 h post-treatment. BmN cells (1.25×10^5^ cells/well) transfected with dsRNA-DsRed (5 μg/well), pIEX-eGFP or treated with ddH_2_O were used as control. The expression of *BmRelish*, *CecA* and *CecB* was quantified by qRT-PCR at 24, 48 and 72 hpi.

### Statistical Analysis

Statistical analysis was performed using GraphPad Prism 8 (GraphPad Software, USA). The statistical significance was represented by P values of >0.05, <0.05, <0.01 or <0.001. All results are presented as mean ± SD from three independent experiments.

## Result

### Chromosomal Location, Subcellular Localization and Phylogenetic Tree Analysis of Silkworm Sirtuin Family Genes

**Figure 1:**
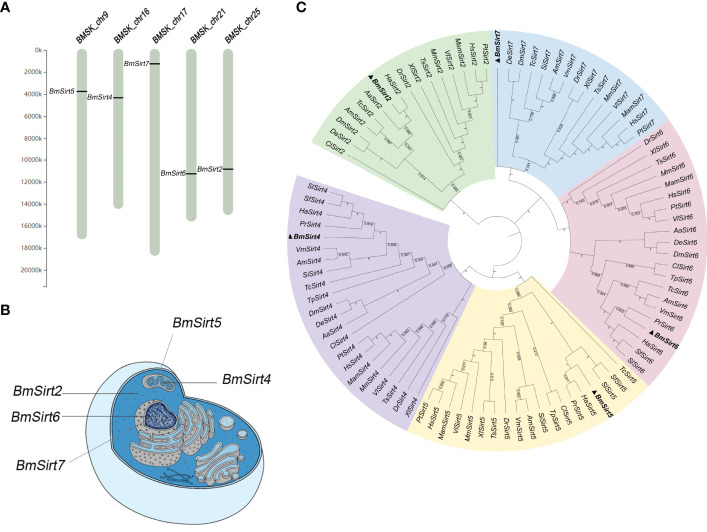
Chromosomal location, subcellular localization and phylogenetic analysis of silkworm members of the Sirtuin Family. **(A)** Chromosomal location of the silkworm *Sirtuin* genes. **(B)** Predicted subcellular localization of silkworm *Sirtuin* genes. **(C)** Phylogenetic analysis of *Sirtuin* family members from the silkworm and other insect, invertebrate and vertebrate species. Bayesian phylogenetic tree was generated based on the Sirtuins nucleotide sequences (The Gene ID of each sequence is shown in **Table S2**). Five branches were color-coded with cyan (Sirt2), purple (Sirt4), yellow (Sirt5), pink (Sirt6) and green (Sirt7). Aa, *A*; *albopictus* Hs, *H*; *sapiens;* Mm, *M*; *musculus;* Dr, *D*; *rerio;* Bm, *B*; *mori;* Dm, *D*; *melanogaster;* Am, *A*; *mellifera;* Tc, *T*; *castaneum;* Xl, *X*; *laevis*, Pt, *P*; *troglodytes*, Mam, *M*; *mulatta*, Ts, *T*; *scripta elegans*, Vl, *V*; *lagopus*, Cl, *C*; *lectularius*, Sf, *S*; *frugiperda*, Ha, *H*; *armigera*, De, *D*; *elegans*, Si, *S*; *invicta*, Vm, *V*; *mandarinia*, Pr, *P*; *rapae*, Tp, *T*; *palmi*, Sl, *S*; *litura*.

The silkworm Sirtuin gene family includes five members named *BmSirt2*, *BmSirt4*, *BmSirt5*, *BmSirt6* and *BmSirt7*. The chromosomal location of silkworm Sirtuins was analyzed according to the silkworm genome database from SilkDB 3.0. *BmSirt2*, *BmSirt4*, *BmSirt5*, *BmSirt6*, and *BmSirt7* were found to be located on chromosome 25, 16, 9, 21 and 17, respectively (**Figure 1A**). The prediction of subcellular localization showed BmSirt2 as a putative cytoplasmic protein (**Figure 1B**). BmSirt4 and BmSirt5 were predicted to be located in the mitochondria, whereas BmSirt6 and BmSirt7 were presumed to be nuclear (**Figure 1B**). In addition, phylogenetic analysis was performed based on the multiple alignment of Sirtuins from different species including mammals, fish, nematodes and insects. Phylogenetic analysis of the nucleotide sequences of Sirtuins showed that BmSirt2 was closely related to *H. armigera* Sirt2. Sirt4 of *B. mori*, *H. armigera*, *P. rapae*, *Spodoptera litura* and *S. frugiperda* were located in the same clade. BmSirt5 was closely related to Sirt5 from *H. armigera* and *P. rapae*. BmSirt6 and *P. rapae* Sirt6 were assigned to a separate clade. However, compared with the Sirt7 of other species, BmSirt7 was found to belong to an independent branch (**Figure 1C**).

### The Response of Silkworm Sirtuin Expression to BmNPV Infection

**Figure 2:**
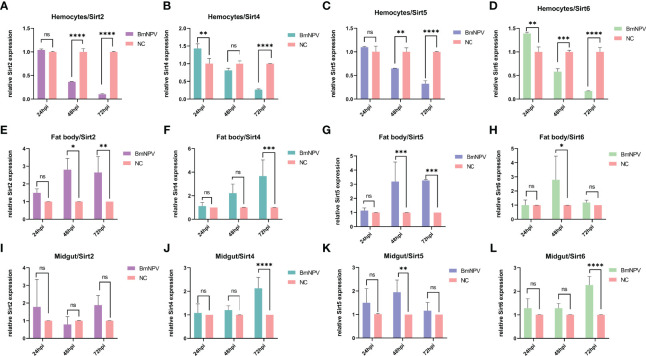
Expression of Sirtuin family genes in hemocytes, fat body and midgut after BmNPV infection in the silkworm. **(A–D)** Detection of the expression of *BmSirt2*, *BmSirt4*, *BmSirt5* and *BmSirt6* in hemocytes. **(E–H)** Transcriptional response of *BmSirt2*, *BmSirt4*, *BmSirt5* and *BmSirt6* to BmNPV infection in fat body. **(I–L)** Detection of the expression of *BmSirt2*, *BmSirt4*, *BmSirt5* and *BmSirt6* in midgut. Negative control represents uninfected larvae. Each bar represents the mean ± SD. *p < 0.05, **p < 0.01, ***p < 0.001, ****p < 0.0001. ns, not significant.

To explore the expression of Sirtuin genes during BmNPV infection in the silkworm, hemocytes, fat body and midgut from BmNPV-infected and uninfected larvae were collected to detect the expression level of their mRNAs by qRT-PCR. In BmNPV-infected hemocytes, the mRNA levels of *BmSirt2*, *BmSirt5*, and *BmSirt6* were significantly decreased at 48 and 72 hpi (**Figures 2A, C, D**). *BmSirt4* expression was significantly down-regulated at 72 hpi and up-regulated at 24 hpi in hemocytes after BmNPV infection (**Figure 2B**). The expression of *BmSirt6* was induced in hemocytes by BmNPV at 24 hpi (**Figure 2D**). The expression of *BmSirt2* and *BmSirt5* were significantly up-regulated at 48 and 72 hpi in the fat body after BmNPV infection (**Figures 2E, G**). *BmSirt4* and *BmSirt6* expression was also significantly induced by BmNPV infection at 72 hpi and 48 hpi, respectively (**Figures 2F, H**). Compared to uninfected midgut, the expression of *BmSirt4* and *BmSirt6* were significantly up-regulated in the BmNPV-infected midgut at 72 hpi (**Figures 2J, L**). *BmSirt5* expression was significantly induced by BmNPV infection in the midgut at 48 hpi (**Figure 2K**). *BmSirt2* in the midgut did not respond to viral infection (**Figure 2I**). The transcription level of *BmSirt7* in fat body, hemocytes and midgut was almost undetectable by RT-PCR which prevented qRT-PCR analysis of its response to the virus infection (**Figure S1**).

### BmSirt2 and BmSirt5 Have Potential Antiviral Functions

**Figure 3:**
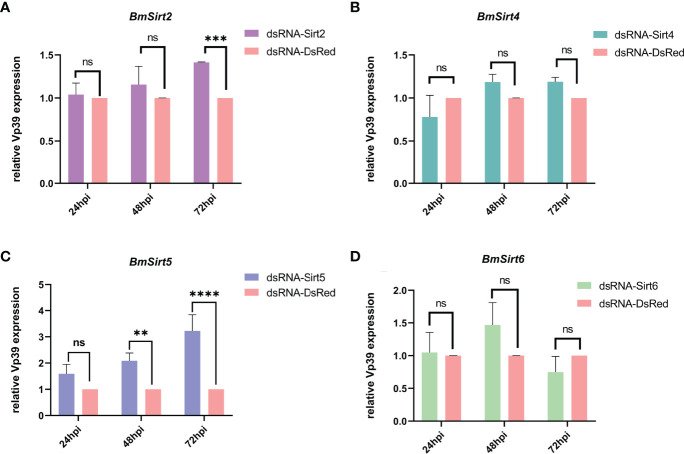
Detection of the expression of the BmNPV capsid gene *Vp39* by qRT-PCR after knockdown of Sirtuin genes in BmN cells. **(A)** The viral gene *Vp39* was significantly up-regulated at 72 hpi after knockdown of *BmSirt2*. **(B)** The expression of *Vp39* did not change significantly after knockdown of *BmSirt4*. **(C)** *Vp39* expression was significantly up-regulated at 48 and 72 hpi after knockdown of *BmSirt5*. **(D)** *Vp39* expression did not change significantly after knockdown of *BmSirt6*. The BmN cells treated with dsRNA-DsRed were used as negative control. Each bar represents the mean ± SD. **p < 0.01, ***p < 0.001, ****p < 0.0001. ns, not significant.

The effect of knockdown of Sirtuin genes on BmNPV replication was further examined in BmN cells. The mRNA levels of *BmSirt2*, *BmSirt4*, *BmSirt5* and *BmSirt6* in BmN cells could be significantly reduced by transfecting the corresponding dsRNAs (**Figure S2**). qRT-PCR results showed that the viral gene *Vp39* was significantly up-regulated (1.4-fold) at 72 hpi when endogenous *BmSirt2* was knocked down (**Figure 3A**). Noteworthily, knockdown of endogenous *BmSirt5* could significantly up-regulate the expression of *Vp39* at both 48 hpi (2.1-fold) and 72 hpi (3.2-fold) (**Figure 3C**). Knockdown of *BmSirt4* and *BmSirt6* had no significant effect on BmNPV replication (**Figures 3B, D**). These results indicated that *BmSirt2* and *BmSirt5* have the potential to inhibit BmNPV replication, with knockdown of *BmSirt5* exhibiting the most potent effects.

### Knockdown of *BmSirt5* Significantly Promoted BmNPV Replication

**Figure 4:**
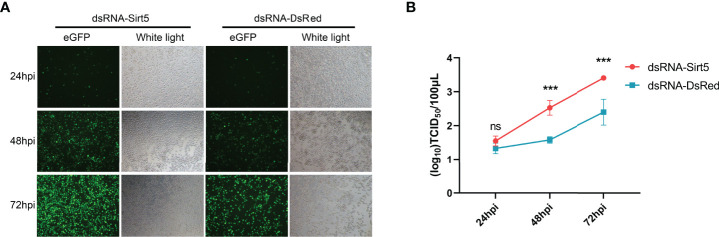
Knockdown of *BmSirt5* significantly promoted BmNPV replication in BmN cells. **(A)** Green fluorescence images of BmNPV-eGFP infected cells observed by an inverted microscope (100×) at 24, 48 and 72 hpi. **(B)** Determination of viral titers in dsRNA-BmSirt5-transfected BmN cells at 24, 48 and 72 hpi. DsRNA of DsRed was used as a negative control. Each bar represents the mean ± SD. ***p < 0.001. ns, not significant.

To further verify the effect of *BmSirt5*, virus titers and GFP expression were determined following BmNPV-eGFP infection in the knockdown condition. The number of green fluorescent cells in dsRNA-BmSirt5-transfected BmN was higher than the control group, and the fluorescence intensity was stronger at 48 and 72 hpi (**Figure 4A**). At 48 and 72 hpi, determination of the TCID_50_ revealed an increased titer in the cell supernatant after knockdown of *BmSirt5* in BmN cells (**Figure 4B**).

### Over-Expression of *BmSirt5* Suppressed BmNPV Replication

**Figure 5:**
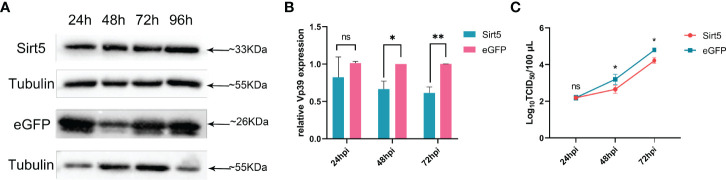
Over-expression of *BmSirt5* inhibited the replication of BmNPV. **(A)** The expression level of exogenous *BmSirt5* in BmN cells was determined by Western blotting with flag antibody at 24, 48, 72 and 96 h post transfection. BmN cells transfected with pIEX-eGFP were used as a negative control. **(B)** Quantification of BmNPV gene *Vp39* expression by qRT-PCR in BmN cells transfected with pIEX-BmSirt5 or pIEX-eGFP at 24, 48 and 72 h after BmNPV infection. **(C)** Viral titer determination using the TCID_50_ assay in supernatants of BmN cells transfected with pIEX-BmSirt5 or pIEX-eGFP at 24, 48 and 72 hpi. Each bar represents the mean ± SD. *p < 0.05, **p < 0.01. ns, not significant.

To increase the expression levels of *BmSirt5*, BmN cells were transfected with the pIEX-BmSirt5 plasmid. Western blotting showed that exogenous *BmSirt5* (detected with Flag antibody) was successfully expressed in BmN cells at 24, 48, 72 and 96 h (**Figure 5A**). BmN cells transfected with pIEX-eGFP were used as control (**Figure 5A**). When cells were infected with BmNPV at MOI 1 at 24 h post-transfection, the expression of the viral gene *Vp39* was significantly down-regulated in the BmSirt5-over-expressing BmN cells at 48 and 72 hpi (**Figure 5B**). Measurement of TCID_50_ showed that the virus titer was significantly decreased at 48 and 72 hpi in BmN cells after over-expression of *BmSirt5* (**Figure 5C**).

### Treatment With the Sirt5 Inhibitor, Suramin, Promoted BmNPV Replication

**Figure 6:**
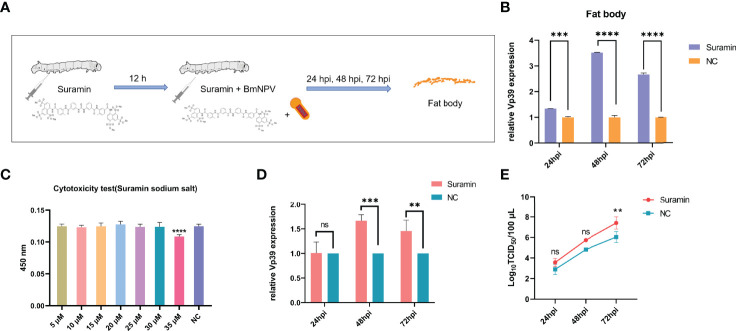
The Sirt5 inhibitor, Suramin, promoted BmNPV replication. **(A)** Schematic diagram of treatment of silkworms with Suramin. **(B)** Quantification of the expression of the BmNPV capsid gene *Vp39* by qRT-PCR after treatment with Suramin in the silkworm fat body at 24, 48 and 72 hpi. **(C)** Cytotoxicity assay of different concentrations of Suramin in BmN cells. **(D)** Quantification of *Vp39* expression by qRT-PCR after treatment with Suramin in BmN cells at 24, 48 and 72 hpi. **(E)** Determination of viral titers using the TCID_50_ assay. Each bar represents the mean ± SD. **p < 0.01, ***p < 0.001, ****p < 0.0001. ns, not significant.

Silkworms were treated with Suramin (3 μg/larva) for 12 h and subsequently infected with BmNPV. Fat body samples were collected at 24, 48 and 72 hpi to detect the expression of *Vp39* by qRT-PCR (**Figure 6A**). **Figure 6B** showed that *Vp39* expression was significantly up-regulated in the fat body of silkworm treated with Suramin at 24, 48 and 72 hpi. These results suggested that treatment with Suramin could promote BmNPV replication in silkworm larvae. To further clarify the effect of *BmSirt5* on BmNPV replication, we also pre-treated BmN cells with Suramin. Following the determination of the cellular toxicity of Suramin, the 30 μM concentration was selected for treatment of BmN cells (**Figure 6C**). *Vp39* expression was significantly up-regulated in the Suramin-treated cells at 48 and 72 hpi (**Figure 6D**). Moreover, virion titers in the supernatant of Suramin-treated cells increased significantly at 72 hpi (**Figure 6E**).

### *BmRelish* Expression Was Induced After BmNPV Infection

**Figure 7:**
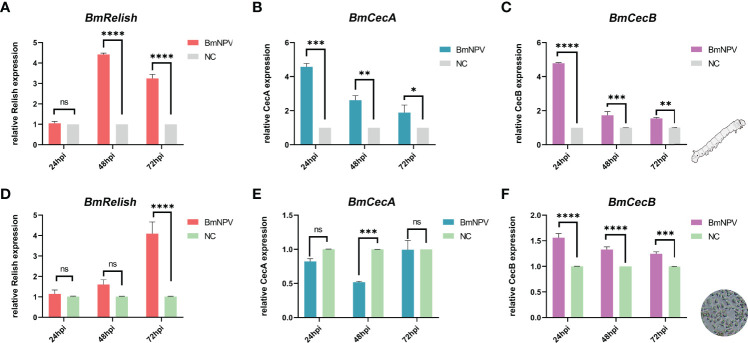
Induction of *BmRelish* expression after BmNPV infection. **(A–C)** Quantification of the expression of *BmRelish*, *CecA* and *CecB* by qRT-PCR in the fat body of virus-infected silkworm larvae. **(D–F)** Quantification of the expression of *BmRelish*, *CecA* and *CecB* in BmN cells by qRT-PCR at 24, 48 and 72 h after BmNPV infection. Negative control corresponds to uninfected samples. Each bar represents the mean ± SD. *p < 0.05, **p < 0.01, ***p < 0.001, ****p < 0.0001. ns, not significant.

Relish is one of the key factors in the silkworm innate immune antiviral pathways that has been shown to be involved in antiviral defense (29). The CecA and CecB antimicrobial peptide genes were selected as downstream factors of Relish-mediated pathway. After BmNPV infection, the expression of *BmRelish* was significantly up-regulated at 48 and 72 hpi (**Figure 7A**), and the expression of both *CecA* and *CecB* was also significantly increased at 24, 48 and 72 hpi in the fat body of virus-infected silkworms (**Figures 7B, C**). In BmN cells, *BmRelish* expression was significantly up-regulated in BmNPV-infected cells at 72 hpi (**Figure 7D**). The expression of *CecB* was significantly induced at 24, 48 and 72 hpi (**Figure 7F**). However, *CecA* expression was decreased at 48 hpi (**Figure 7E**). These results indicated that BmRelish-mediated pathways could respond to BmNPV infection by increased expression of antimicrobial peptide genes.

### *BmSirt5* Could Enhance BmRelish-Mediated Pathways During BmNPV Infection

**Figure 8:**
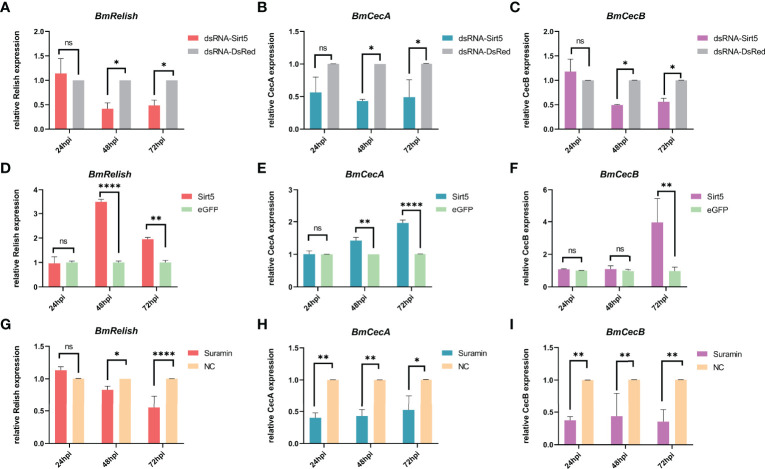
Enhancement of BmRelish-mediated immunity by *BmSirt5* during BmNPV infection. **(A–C)** Quantification of the expression of *BmRelish*, *CecA* and *CecB* by qRT-PCR after knockdown of *BmSirt*5 in BmN cells at 24, 48 and 72 h after BmNPV infection. BmN cells treated with dsRNA-DsRed were used as negative control. **(D–F)** Quantification of the expression of *BmRelish*, *CecA* and *CecB* by qRT-PCR after over-expression of *BmSirt5* in BmN cells at 24, 48 and 72 hpi. BmN cells treated with pIEX-eGFP were used as negative control. **(G–I)** Quantification of the expression of *BmRelish*, *CecA* and *CecB* by qRT-PCR after treatment with Suramin in BmN cells. BmN cells treated with ddH_2_O were used as negative control. Each bar represents the mean ± SD. *p < 0.05, **p < 0.01, ****p < 0.0001. ns, not significant.

To explore the effect of *BmSirt5* on the activation of BmRelish-mediated immunity, BmN cells transfected with dsRNA-BmSirt5 (5 μg/well), pIEX-BmSirt5 plasmid (500 ng/well) or treated with Suramin (30 μM) were infected with BmNPV-eGFP at 1 MOI after 24 h post treatment. The expression of *BmRelish*, *CecA* and *CecB* was significantly down-regulated at 48 hpi and 72 hpi after knockdown of *BmSirt5* (**Figures 8A–C**). After over-expression of *BmSirt5*, the expression of *BmRelish* and *CecA* was significantly up-regulated at 48 and 72 h after BmNPV infection (**Figures 8D, E**), and *BmCecB* expression was significantly up-regulated at 72 hpi (**Figure 8F**). After treatment of BmN cells with Suramin, it was observed that the expression of *BmRelish* was significantly down-regulated at 48 and 72 hpi (**Figure 8G**), and the expression of both *CecA* and *CecB* was significantly down-regulated at 24, 48 and 72 hpi (**Figures 8H, I**). These results suggest that *BmSirt5* could enhance BmRelish-mediated immunity against BmNPV infection.

## Discussion

Sirtuins represent a class of NAD^+^-dependent Lysine deacetylases with complex functions in cellular physiology. Mammalian Sirtuins play an important role in maintaining a well-established metabolic pathway network. Much effort was devoted into exploring their roles in human cancers and aging (2). However, a growing literature in virus research brings these enzymes into focus as antiviral factors (3). In particular, some progress has been made in the study of the involvement of Sirtuin family genes in the immune response to viruses in mammals (6, 7, 30, 31).

Viruses need to rely on host metabolism to obtain the energy and building blocks to meet their replication needs. The various metabolic regulatory functions of Sirtuins may be the basis for their regulation of viral immune responses. Studies have shown that the de-acetylase activity of Sirt3 was a key factor in inhibiting the production of HCMV (10). Overexpression of Sirt2 type 5 isoenzyme inhibited HBV replication (9). Conversely, Sirt1 knockdown promoted Kaposi’s sarcoma-associated herpesvirus replication (32). Sirtuin inhibition also has been shown to promote influenza A virus replication (33).

In this study, knockdown of *BmSirt4* and *BmSirt6* has no significant effect on BmNPV replication. However, BmNPV replication was promoted at 72 hpi when endogenous *BmSirt2* was knocked down. Noteworthily, knockdown of endogenous *BmSirt5* could promote BmNPV replication at both 48 hpi and 72 hpi. Therefore, *BmSirt5* was more extensively investigated for its antiviral mechanism in this study. The role of *BmSirt2* in virus infection of silkworm is also worthy of further study, which will be the subject of future research. Here, we demonstrated that *BmSirt5* could inhibit BmNPV replication by different approaches (over-expression, knock down, enzymatic inhibition) and assays (qRT-PCR, green fluorescence, virus titer assay) both *in vivo* (silkworm larvae) and *in vitro* (BmN cells). Mechanistically, our preliminary findings indicated that *BmSirt5* could enhance BmRelish-mediated immune pathways during BmNPV infection. In a mammalian system, it was also shown that Sirt5 could compete with Sirt2 to interact with p65 in a de-acetylase activity-independent way to block the de-acetylation of p65 by Sirt2, consequently leading to the increased acetylation of p65 and activation of NF-κB (34). The antiviral defense of silkworms mainly relies on innate immunity pathways such as RNA interference (RNAi), NF-κB-mediated defense mechanisms and the Immune deficiency (Imd) and Stimulator of Interferon Gene (STING) pathways (29, 35). While RNAi is considered the most important antiviral pathway (29), the NF-κB-mediated and STING pathways also play a critical role in the outcome of viral infections in the silkworm (29). Recent studies have elucidated a STING-mediated antiviral pathway in the silkworm (36) in which production of cyclic guanosine monophosphate–adenosine monophosphate (cGAMP) was triggered upon BmNPV infection. As a secondary messenger molecule, cGAMP induced the activation of BmSTING that resulted in cleavage and nuclear translocation of BmRelish to stimulate the transcription of antimicrobial and antiviral genes (36). Our study revealed that *BmSirt5* may promote the activation of *BmRelish* through a specific mechanism, leading to the enhanced transcription of downstream antiviral factors (29). However, the mechanism by which BmSirt5 can regulate the activity of BmRelish remains to be elucidated. Nevertheless, we demonstrated that treatment of silkworm larvae and BmN cells with Suramin resulted in enhanced BmNPV replication. Suramin has been shown to inhibit Sirt5 NAD^+^-dependent de-acetylase activity (28) and it is therefore suggested that BmSirt5 plays its antiviral role through this enzymatic activity. In the future, we will further study the specific mechanism of *BmSirt5* regulation of downstream genes through activation of BmRelish, so as to provide new insights into this specific pathway of antiviral defense in *Bombyx*.

**Figure 9:**
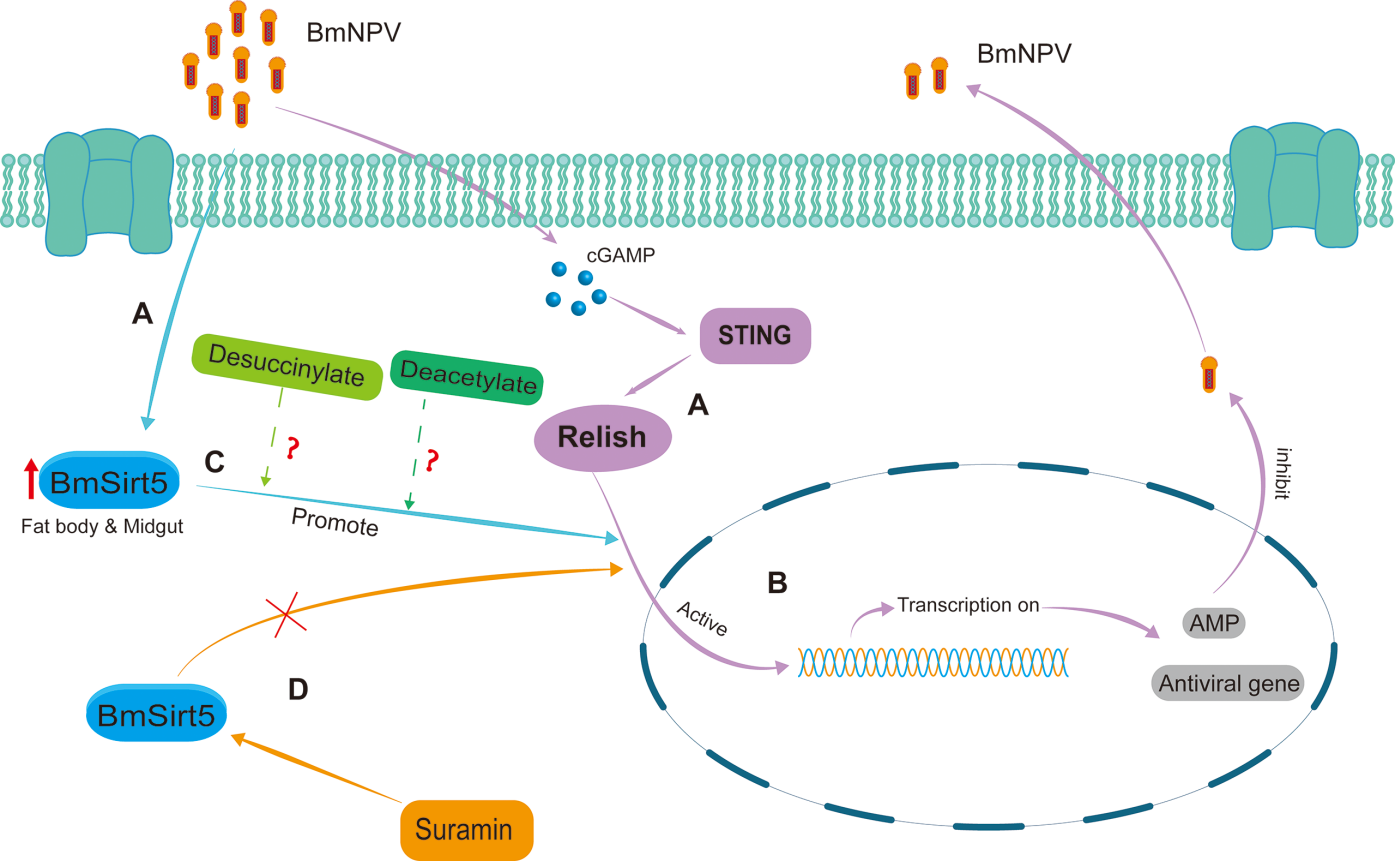
Schematic diagram of the putative antiviral mechanism by which BmSirt5 inhibited BmNPV proliferation in the silkworm. **(A)** *BmSirt5* expression was significantly induced by BmNPV infection in the fat body or midgut, together with the activation of other host antiviral pathways such as STING. **(B)** The innate immune pathway mediated by BmRelish can induce the production of downstream antimicrobial peptide (AMP) genes and other antiviral genes to resist BmNPV infection. **(C)** Nuclear translocation of Relish may be stimulated by de-succinylation or de-acetylation activities of BmSirt5. **(D)** Inhibition of the NAD^+^-dependent de-acetylase activity of *BmSirt5* by Suramin could interfere with the activation of BmRelish, for instance by inhibition of its transfer to the nucleus.

Based on the results of this study, the possible antiviral mode of action of BmSirt5 in the silkworm can be summarized as follows (**Figure 9**). In BmNPV-infected silkworms, *BmSirt5* expression becomes significantly induced in the fat body or midgut tissue while other reports also documented the activation of the STING pathway (**Figure 9A**). The antiviral pathway mediated by BmRelish is known to induce the production of downstream antimicrobial peptides and other (to be identified) antiviral effectors to resist BmNPV infection (29) (**Figure 9B**). *BmSirt5* potentially could enhance BmRelish function by its de-succinylation or de-acetylation activities (**Figure 9C**). By inhibiting the NAD^+^-dependent de-acetylase activity of BmSirt5, Suramin may reduce cleavage or nuclear entry of BmRelish which is required for the induction of innate immune genes (**Figure 9D**).

## Data Availability Statement

The original contributions presented in the study are included in the article/**Supplementary Material**. Further inquiries can be directed to the corresponding authors.

## Author Contributions

MZ and SF did the experiments, collected and analyzed data and drafted the manuscript. JX, YW, HW, XL, and YG helped with sample preparation and data analysis. LS revised the manuscript. JS participated in coordination of the study, and revised the manuscript. MF designed the experimental scheme, collected and analyzed data and revised the manuscript. All authors read and approved the final manuscript.

## Funding

This work was supported by the Basic and Applied Basic Research Project of Guangzhou Basic Research Program (2022, Project Title: Functional study on the regulation of BmNPV replication by the silkworm Sirtuin gene family), The Natural Science Foundation of Guangdong Basic and Applied Basic Research Fund (2022A1515012657); South China Agricultural University high-level talent launch project; and Guangdong Provincial Promotion Project on Preservation and Utilization of Local Breed of Livestock and Poultry (No.2018-143).

## Conflict of Interest

The authors declare that the research was conducted in the absence of any commercial or financial relationships that could be construed as a potential conflict of interest.

## Publisher’s Note

All claims expressed in this article are solely those of the authors and do not necessarily represent those of their affiliated organizations, or those of the publisher, the editors and the reviewers. Any product that may be evaluated in this article, or claim that may be made by its manufacturer, is not guaranteed or endorsed by the publisher.

## Supplementary Material

Supplementary Figure 1 Quantification of BmSirt7 expression by RT-PCR in the hemocytes, fat body and midgut. **(A)** Expression of *BmSirt7* in the uninfected silkworm. M: molecular weight marker (DL5000). **(B)** Expression of *BmSirt7* in the BmNPV-infected silkworm. M: molecular weight marker (DL2000). The *Rp49* gene was used as reference gene.

Supplementary Figure 2 Sirtuin expression after specific knockdown in BmN cells at 24 h, 48 h, 72 h and 96 h. **(A)** *BmSirt2*, **(B)** *BmSirt4*, **(C)** *BmSirt5* and **(D)** *BmSirt6*. Each bar represents the mean ± SD. **p < 0.01, ***p < 0.001, ****p < 0.0001.
