# Supplementary figures and images for: Sirt5 Inhibits BmNPV Replication by Promoting a Relish-Mediated Antiviral Pathway in Bombyx mori
Source: Front Immunol. 2022 May 23;13:906738. doi: 10.3389/fimmu.2022.906738 (PMC9186105; doi:10.3389/fimmu.2022.906738)

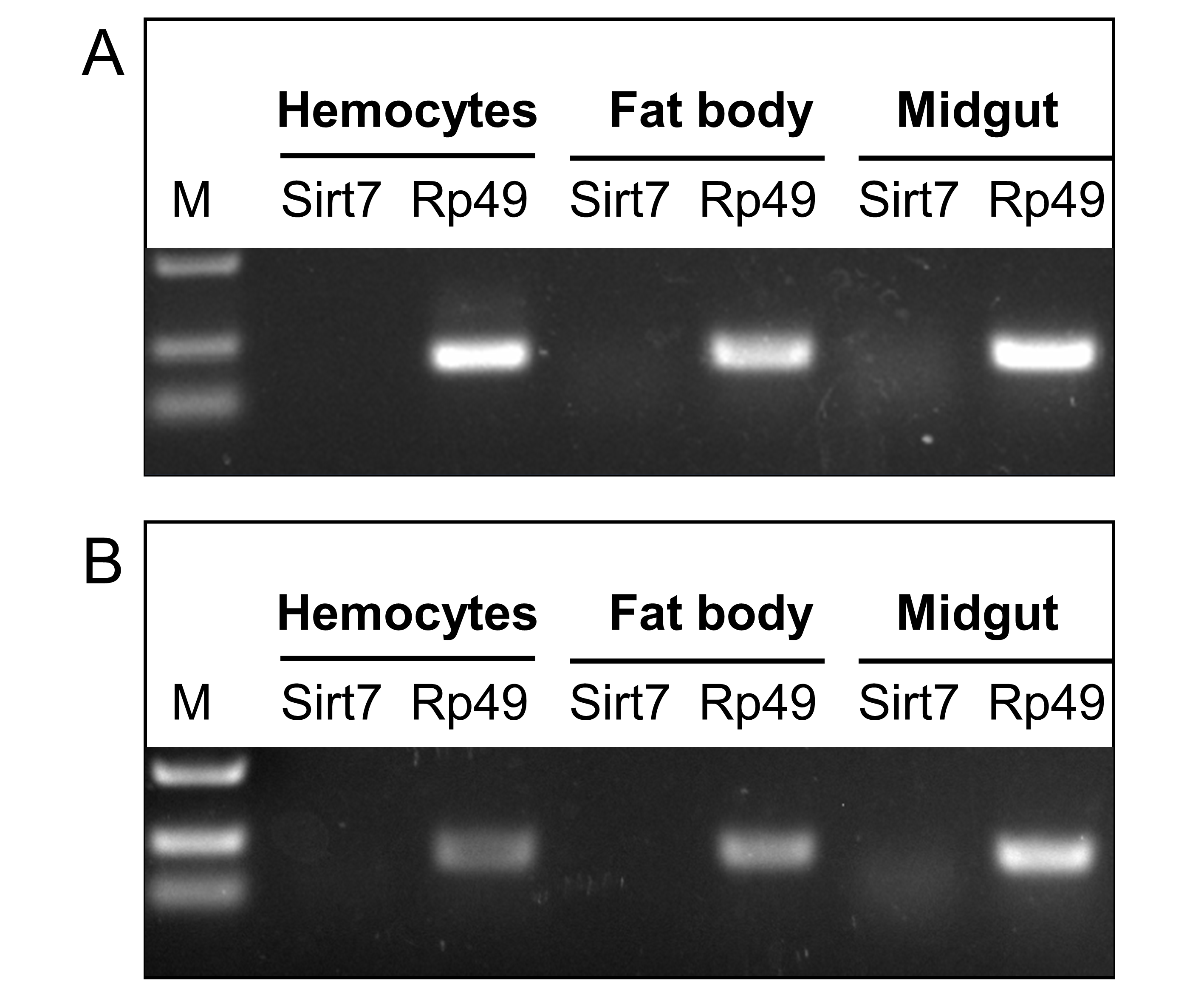

Supplement: Supplementary Figure 1 — Quantification of BmSirt7 expression by RT-PCR in the hemocytes, fat body and midgut. (A) Expression of BmSirt7 in the uninfected silkworm. M: molecular weight marker (DL5000). (B) Expression of BmSirt7 in the BmNPV-infected silkworm. M: molecular weight marker (DL2000). The Rp49 gene was used as reference gene. [file Image_1.tif]

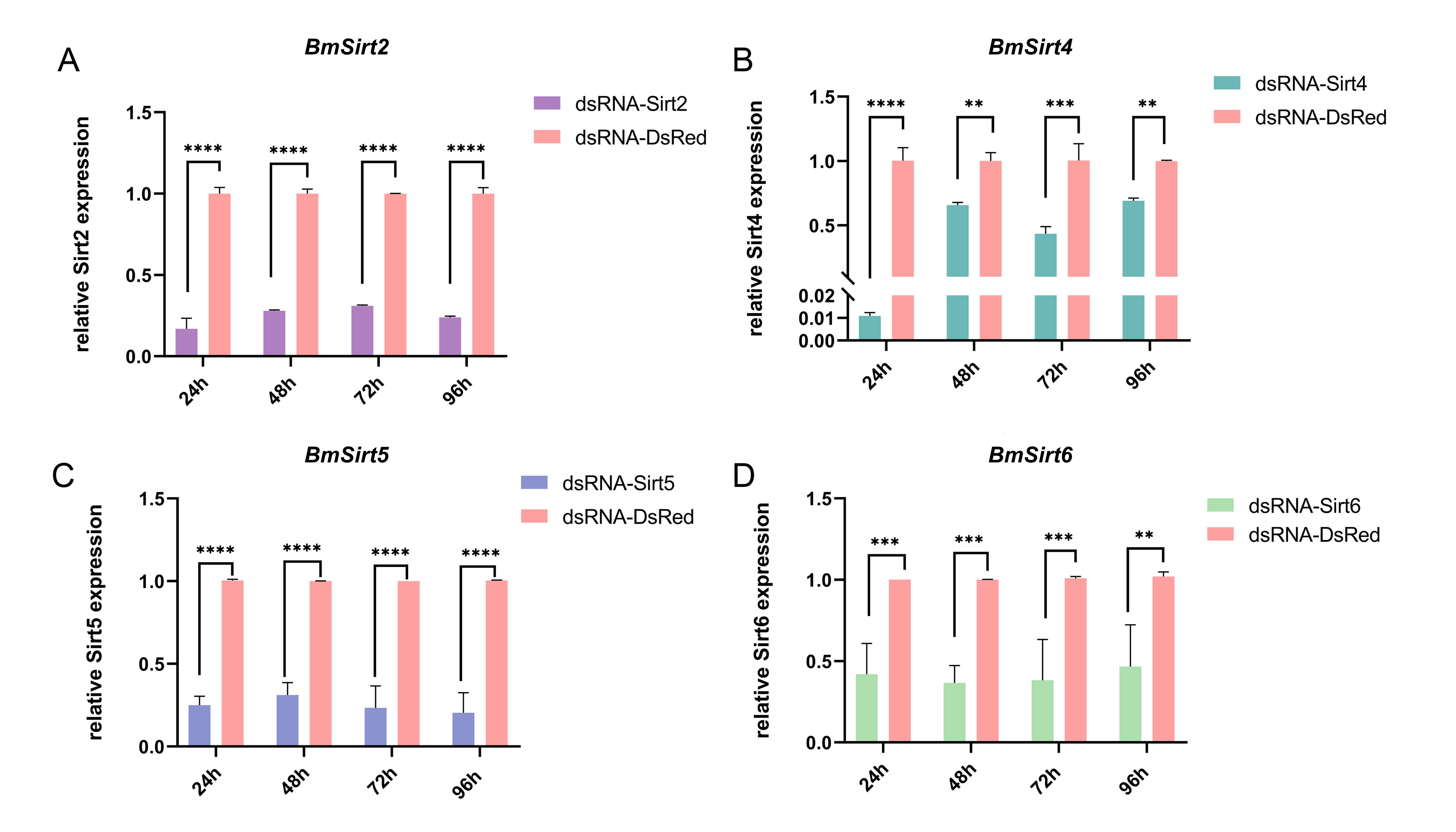

Supplement: Supplementary Figure 2 — Sirtuin expression after specific knockdown in BmN cells at 24 h, 48 h, 72 h and 96 h. (A) BmSirt2, (B) BmSirt4, (C) BmSirt5 and (D) BmSirt6. Each bar represents the mean ± SD. **p < 0.01, ***p < 0.001, ****p < 0.0001. [file Image_2.tif]
