# Supplementary material for: Sirt5 Inhibits BmNPV Replication by Promoting a Relish-Mediated Antiviral Pathway in Bombyx mori
Source: Front Immunol. 2022 May 23;13:906738. doi: 10.3389/fimmu.2022.906738 (PMC9186105; doi:10.3389/fimmu.2022.906738)
Supplement: Supplementary file 3 [file Table_1.docx]

Table S1 Primers used in the study.

| Purpose | Gene name | Sequence of Oligonucleotide (5’-3’) |
| --- | --- | --- |
| RNAi  (dsRNA) | BmSirt2 | F: taatacgactcactatagggAACTACCTCAACCACAGGCG |
|  |  | R: taatacgactcactatagggTGATGCAAATGGTTGCACTT |
|  | BmSirt4 | F: taatacgactcactatagggTTCCGAAACATAAACCACCC |
|  |  | R: taatacgactcactatagggTTTCAAAGGCCCTTCACACT |
|  | BmSirt5 | F: taatacgactcactatagggGGAGGGCTTTGGAGGAAATA |
|  |  | R: taatacgactcactatagggCATTGCATGTTCAGCTGCTT |
|  | BmSirt6 | F: taatacgactcactatagggCACACTGGAGCTGGCATAAG |
|  |  | R: taatacgactcactatagggAATGCCATTCTGCCATTAGC |
|  | DsRed | F: taatacgactcactatagggGAAGCTGAAGGTGACCAAGG |
|  |  | R: taatacgactcactatagggTGGTGTAGTCCTCGTTGTGG |
| qRT-PCR | BmSirt2 | F: ATTCGCCTCCCGGAAAAAGT |
|  |  | R: AGTTGTTGGCGGGACATTCT |
|  | BmSirt4 | F: TCGCGTGGAACAAGTCAGAA |
|  |  | R: AAACTGTGAGGCTTGATCCCA |
|  | BmSirt5 | F: TCAGTGGTATATCCTGCGGC |
|  |  | R: GTGGCCGGTGTTGGTTCTAT |
|  | BmSirt6 | F: TGAGCATATGTCTAGGTACAACACT |
|  |  | R: CATGTTTTGTTGGTTGTAGGTTGC |
|  | Rp49 | F: CAGGCGGTTCAAGGGTCAATAC |
|  |  | R: TGCTGGGCTCTTTCCACGA |
|  | Vp39 | F: CTAATGCCCGTGGGTATGG |
|  |  | R: TTGATGAGGTGGCTGTTGC |
|  | BmRelish | F: TTCGGTGGAATGGGTATCAT |
|  |  | R: GCTGAACTTCAAACGCACAA |
|  | BmCecA | F: GCCCAGGTGGAAACTCTTCA |
|  |  | R: GCTTGCCCTATGACGGCTAT |
|  | BmCecB | F: ATCCTTCGTCTTCGCTCTGG |
|  |  | R: ACGGATGTTCCTGCCCATTT |
| RT-PCR | BmSirt7 | F: ACACTGATGCATCTTCCCCA |
|  |  | R: AACCGATCTGCTAGCCTCCT |
| over-  expression | BmSirt5 | F: GAGCTCATGTACCCATACGACGTCCCAGACTACGCTAGTTCAAGACAAT |
|  |  | R: GTCGACCTACTTATCGTCGTCATCCTTGTAATCGATCTTATCGTCGTCATCCTTGTAATCTCCCTTATCGTCGTCATCCTTGTAATCATCAGCCAGTGCTTGTGG |

The underline represents *Sac* I and *Sal* I.

The wavy line represents the sequence of 3×Flag tag.
