# Supplementary material for: Sirt5 Inhibits BmNPV Replication by Promoting a Relish-Mediated Antiviral Pathway in Bombyx mori
Source: Front Immunol. 2022 May 23;13:906738. doi: 10.3389/fimmu.2022.906738 (PMC9186105; doi:10.3389/fimmu.2022.906738)
Supplement: Supplementary file 4 [file Table_2.doc]

Table S2 The information of Sirtuins used for phylogenetic analysis.

| **Genes** | **ID** | **Genes** | **ID** | **Genes** | **ID** |
| --- | --- | --- | --- | --- | --- |
| PtSirt2 | XM_011159443.3 | HsSirt4 | NM_001385733.1 | VmSirt6 | XM_035871297.1 |
| HsSirt2 | AY030277.1 | MamSirt4 | XM_015152957.2 | AmSirt6 | XM_396298.7 |
| MamSirt2 | XM_015123871.2 | MmSirt4 | NM_001167691.1 | TcSirt6 | XM_968003.3 |
| VlSirt2 | XM_041735964.1 | VlSirt4 | XM_041729279.1 | TpSirt6 | XM_034383483.1 |
| MmSirt2 | NM_001122765.1 | TsSirt4 | XM_034791092.1 | ClSirt6 | XM_014388936.2 |
| TsSirt2 | XM_034792321.1 | DrSirt4 | NM_001005988.1 | DmSirt6 | NM_141733.3 |
| XlSirt2 | NM_001095167.1 | XlSirt4 | XM_018243881.2 | DeSirt6 | XM_017263676.1 |
| DrSirt2 | NM_199596.1 | PtSirt5 | XM_009450542.3 | AaSirt6 | XM_019699072.2 |
| HaSirt2 | KY363351.1 | HsSirt5 | NM_001193267.2 | VlSirt6 | XM_041763672.1 |
| BmSirt2 | BMSK0014938 | MamSirt5 | XM_015135582.2 | PtSirt6 | XM_022273576.1 |
| AaSirt2 | XM_019708403.2 | VlSirt5 | XM_041772689.1 | HsSirt6 | NM_001193285.3 |
| TcSirt2 | XM_963962.3 | MmSirt5 | NM_178848.3 | MamSirt6 | XM_002801029.3 |
| AmSirt2 | XM_393038.7 | XlSirt5 | NM_001095497.1 | MmSirt6 | NM_001163430.2 |
| DmSirt2 | NM_001300493.1 | TsSirt5 | XM_034762378.1 | TsSirt6 | XM_034755384.1 |
| DeSirt2 | XM_017258513.1 | DrSirt5 | NM_001002605.1 | XlSirt6 | NM_001092123.1 |
| ClSirt2 | XM_014391605.2 | VmSirt5 | XM_035879689.1 | DrSirt6 | NM_001002071.1 |
| Slsirt4 | XM_022975616.1 | AmSirt5 | XM_625077.6 | PtSirt7 | XM_009433530.3 |
| SfSirt4 | XM_035592319.1 | SiSirt5 | XM_039450238.1 | HsSirt7 | NM_016538.3 |
| HaSirt4 | XM_021326737.1 | TpSirt5 | XM_034376225.1 | MamSirt7 | NM_001257960.1 |
| PrSirt4 | XM_022256791.1 | ClSirt5 | XM_014392519.2 | VlSirt7 | XM_041725174.1 |
| BmSirt4 | BMSK0009272 | PrSirt5 | XM_022264887.1 | MmSirt7 | NM_001363439.1 |
| VmSirt4 | XM_035867325.1 | HaSirt5 | XM_021337284.1 | TsSirt7 | XM_034789856.1 |
| AmSirt4 | XM_623651.6 | BmSirt5 | BMSK0004855 | DrSirt7 | XM_001336402.6 |
| SiSirt4 | XM_011171013.3 | SlSirt5 | XM_022981014.1 | VmSirt7 | XM_035888027.1 |
| TcSirt4 | XM_967874.3 | SfSirt5 | XM_035582862.1 | AmSirt7 | XM_397168.6 |
| TpSirt4 | XM_034375435.1 | TcSirt5 | XM_967725.3 | SiSirt7 | XM_011159443.3 |
| DmSirt4 | NM_132013.3 | SlSirt6 | XM_022968875.1 | TcSirt7 | XM_965249.3 |
| DeSirt4 | XM_017257287.2 | SfSirt6 | XM_035591637.1 | DmSirt7 | NM_143407.3 |
| AaSirt4 | XM_019676138.2 | HaSirt6 | XM_021345441.1 | DeSirt7 | XM_017273644.2 |
| ClSirt4 | XM_014389576.2 | BmSirt6 | BMSK0012256 | BmSirt7 | BMSK0009754 |
| PtSirt4 | XM_009426390.3 | PrSirt6 | XM_022273576.1 |  |  |
